# Cadmium specific proteomic responses of a highly resistant *Pseudomonas aeruginosa* san ai†

**DOI:** 10.1039/c8ra00371h

**Published:** 2018-03-16

**Authors:** Lidija Izrael-Živković, Milena Rikalović, Gordana Gojgić-Cvijović, Saša Kazazić, Miroslav Vrvić, Ilija Brčeski, Vladimir Beškoski, Branka Lončarević, Kristina Gopčević, Ivanka Karadžić

**Affiliations:** Department of Chemistry, Faculty of Medicine, University of Belgrade Visegradska 26 Belgrade Serbia ivanka.karadzic@med.bg.ac.rs +381-113607067; Faculty of Applied Ecology Futura, University of Singidunum Belgrade Serbia; Institute of Chemistry, Technology and Metallurgy, Department of Chemistry, University of Belgrade Studentski trg 12 Belgrade Serbia; Ruđer Bošković Institute Bijenička cesta 54 Zagreb Croatia; Faculty of Chemistry, University of Belgrade Studentski trg 12-16 Belgrade Serbia

## Abstract

*Pseudomonas aeruginosa* san ai is a promising candidate for bioremediation of cadmium pollution, as it resists a high concentration of up to 7.2 mM of cadmium. Leaving biomass of *P. aeruginosa* san ai exposed to cadmium has a large biosorption potential, implying its capacity to extract heavy metal from contaminated medium. In the present study, we investigated tolerance and accumulation of cadmium on protein level by shotgun proteomics approach based on liquid chromatography and tandem mass spectrometry coupled with bioinformatics to identify proteins. Size exclusion chromatography was used for protein prefractionation to preserve native forms of metalloproteins and protein complexes. Using this approach a total of 60 proteins were observed as up-regulated in cadmium-amended culture. Almost a third of the total numbers of up-regulated were metalloproteins. Particularly interesting are denitrification proteins which are over expressed but not active, suggesting their protective role in conditions of heavy metal exposure. *P. aeruginosa* san ai developed a complex mechanism to adapt to cadmium, based on: extracellular biosorption, bioaccumulation, the formation of biofilm, controlled siderophore production, enhanced respiration and modified protein profile. An increased abundance of proteins involved in: cell energy metabolism, including denitrification proteins; amino acid metabolism; cell motility and posttranslational modifications, primarily based on thiol-disulfide exchange, were observed. Enhanced oxygen consumption of biomass in cadmium-amended culture *versus* control was found. Our results signify that *P. aeruginosa* san ai is naturally well equipped to overcome and survive high doses of cadmium and, as such, has a great potential for application in bioremediation of cadmium polluted sites.

## Abstract 2

When exposed to cadmium a highly resistant strain *P. aeruginosa* san ai responds by an increased metalloprotein expression (particularly denitrification proteins), an enhanced respiration, and a pronounced thiol-disulfide protein modifications.

## Graphical abstract

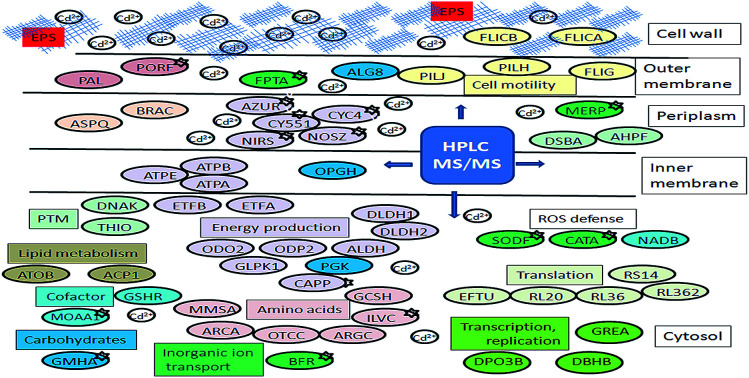

## 1. Introduction

In order to survive heavy metal exposure some microorganisms evolved specific mechanisms of resistance that make them key players in bioremediation strategies.^1^ The use of resistant microbes in the remediation of heavy metals contamination is efficient, relatively inexpensive and reliable.^1,2^ Basically, the mechanisms allowing the microorganisms to cope with metals in the environment are complexation and active efflux of heavy metals. *Pseudomonas* is well-known for their capacity to tolerate high concentrations of several heavy metals including a highly toxic cadmium.^3–5^ Recent reports have highlighted the potential of new isolates of *P. aeruginosa* for application in bioremediation of water and soil contaminated with cadmium.^6,7^ To tolerate heavy metals, *Pseudomonas* demonstrates numerous survival adaptations such as: energy-dependent efflux of metal ions, extracellular biosorption and cellular sequestration, biofilm formation, resistance to oxidative stress, and preservation of iron acquisition and homeostasis.^2–4,8–13^

The key to our understanding of adaptation strategies and mechanisms of *Pseudomonas* heavy metal resistance lies in proteomic changes, which correlate with metabolic paths enabling microbes to survive and persist in heavy metal contaminated environments. However, the most common proteomic studies of *Pseudomonas* strains are related to zinc, copper^14^ and chromium,^8^ while the studies of proteomic fluctuations in response to cadmium are rare and data show unclear and complex responses.^9,11,12^ Comparative proteomics is a powerful technique to identify alterations in protein expression caused by changing environmental conditions and it was used to determine the molecular mechanisms of cadmium (Cd) tolerance in a *Pseudomonas* exposed to cadmium.^8,9,11,12^ In order to enrich metalloproteins in an analyzed proteome, size exclusion chromatography (SEC) has been used for protein prefractionation.^15^ SEC has been reliable in separation of metalloproteins and protein complexes maintaining them in a native state.^15,16^

*P. aeruginosa* san ai, an environmental isolate from mineral cutting oil, was previously used in various studies in our laboratory related to rhamnolipid,^17^ exopolysaccharide (EPS),^18^ the extracellular enzymes, lipase and protease,^19,20^ and their application in detergents^21^ as well as in the study related to the organism's capacity for chromium removal.^22^ In this study, we investigated the distribution of cadmium, its uptake by extracellular polymers and effects of intracellular cadmium on protein expression in *P. aeruginosa* san ai. We used a global proteomic approach based on SEC protein prefractionation coupled with tandem mass spectrometry and bioinformatics to identify specific protein response to cadmium. Proteomics technique was already successfully applied to *P. putida*,^2,9^*P. fluorescens*,^11,12^ and *P. brassicacearum*, but never before to any environmental isolate of *P. aeruginosa*. Furthermore, in order to understand the complex mechanism of response to cadmium, besides proteins we have analyzed the content of: exopolysaccharide, siderophores, nitrate and nitrite, as well the rates of O_2_ consumption.

## 2. Material and methods

### Growth conditions of *P. aeruginosa* san ai

*P. aeruginosa* san ai was isolated from industrial mineral metal-cutting oil.^19^ The nucleotide sequences of 16S rDNA of *P. aeruginosa* san has been submitted in GenBank with accession number JQ012798. Microorganism was deposited in the National Collection of Agricultural and Industrial Microorganisms (NCAIM), Faculty of Food Sciences, Corvinus University of Budapest, Budapest, Hungary, under the label NCAIM (P) B 001380. Microorganism is available from the corresponding author. Genome sequencing and assembly is deposited as NCBI BioProject, accession: PRJNA195719.

*P. aeruginosa* san ai was activated on nutrient agar (Torlak, Belgrade, Serbia) at 30 °C for 24 h and transferred to a 500 mL Erlenmeyer flask, containing 100 mL of Luria broth (LB) medium, g L^−1^ (NaCl – 5, yeast extract – 5 and tryptone – 10) to obtain a preculture. The flask was incubated at 30 °C for 20 hours and shaken at 250 cycles per min with a horizontal shaker (Kuhner, Basel, Switzerland). Actively grown preculture (1 mL) was used to inoculate 100 mL of LB with cadmium and control in 500 mL Erlenmeyer flasks to obtain initial cellular concentrations of approximately 5 × 10^5^ cells per mL and agitated at 250 cycles per min with Kuhner horizontal shaker. LB medium with increasing concentrations of cadmium (mM): 0.45, 0.9, 1.8, 2.7, 3.6, 4.5, 5.4, 6.3 and 7.2, at pH 7.2 was prepared using CdCl_2_·H_2_O stock solution, concentration of 100 mM. Two controls were utilized: non-inoculated controls – LB with appropriate concentrations of cadmium but no *P. aeruginosa* san ai and control of bacterial growth – LB inoculated with *P. aeruginosa* san ai but without cadmium. Bacterial growth was monitored in cadmium supplemented broth and control, as a change of optical density at 580 nm using spectrophotometer Shimadzu UV-2600 (Kyoto, Japan). Measurements of OD_580_ were conducted in triplicate.

Culture broth was centrifuged at 4000 × *g* for 20 min (Sorvall, rotor SS-1, New Town, USA) and the biomass and supernatant obtained were further analyzed.

All spectrophotometric measurements were done on Shimadzu UV-2600 (Kyoto, Japan).

### Evaluation of cadmium resistance

The minimal inhibitory concentration (MIC) of cadmium was determined in LB medium supplemented with cadmium concentrations ranging from 0.5 to 10 mM. Tubes containing growth medium and increasing concentrations of cadmium were inoculated with 24 h culture to obtain an initial optical density of approximately 0.06 at 580 nm. Turbidity was measured after 48 h of growth. The minimum concentration of the metal inhibiting complete growth was taken as the MIC.^23^

### Cadmium determination and distribution

Cadmium was quantified in: biomass, culture supernatant, EPS, cell homogenate and cell debris after homogenization (see below) spectrophotometrically using an iCAP 6500 Duo ICP (Thermo Fisher Scientific, Cambridge, UK) at 214.438 nm.^24^ Biomass, culture supernatant, EPS, homogenate and cell debris were digested with HNO_3_ and H_2_O_2_ in a digester DS-6, Tecator, (Sweden) prior to analysis of cadmium content. Cadmium content in biomass and supernatant was analyzed every 24 h, while EPS, homogenate and cell debris were analyzed after 48 h of bacterial growth. Eluates from gel size exclusion chromatography were measured directly, without prior digestion of the samples.^25^ The amount of cadmium removed by biosorbent at time *t*, was calculated according to:^26^
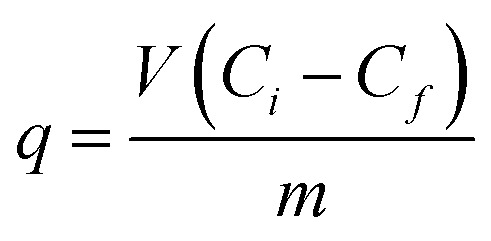
where *q* is cadmium uptake (mM g^−1^), *V* – sample volume (L), *C*_i_ – initial cadmium concentration (mM), *C*_f_ – final cadmium concentration (mM), *m* – mass of dry biosorbent (g)

### Nitrate and nitrite determination

Nitrate and nitrite levels in culture medium were determined by ion chromatography on an Ionchrom analyzer (Dionex, Sunnyvale, USA) with conductivity detector (Dionex, Sunnyvale, USA), SRS controller and autosampler AS 3000 (Thermo Scientific, USA), column Dionex IonPac TM AS14A (4 × 250 mm), eluent: 8.0 mM Na_2_CO_3_/1 mM NaHCO_3_, flow rate: 1 mL min^−1^, applied current: 43 mA, injection volume: 50 μL, standard mix solution of: fluoride, chloride, bromide, nitrite, nitrate, phosphate, sulfate, concentration 0.01 g L^−1^.^27^

### Siderophore determination

Pyoverdin was monitored in culture supernatant by measuring the absorbance at 400 nm. The concentration was calculated using a molar absorption coefficient *ε* (400) = 2 × 10^4^ M^−1^ cm^−1^.^28^ The concentration of pyochelin was determined spectrophotometrically at 310 nm according to Sokol and calculated by *ε* (310) = 4.2 × 10^3^ M^−1^ cm^−1^.^29^ Determinations were conducted in triplicate.

### Exopolysaccharide determination

Exopolysaccharide was measured in culture supernatant after precipitation with three volumes of cold ethanol. EPS was collected by centrifugation at 5000 × *g* for 20 min, air-dried for 24 hours and quantified by measuring mass.^30^

### Respiration measurement

Respiration activity of *P. aeruginosa* san ai was measured using a twelve-channel Micro-Oxymax® respirometer (Columbus Instruments, Columbus, USA) connected to a PC. The experiments were performed in MicroOxymax light-proof 500 mL bottles (Duran, Wertheim, Germany) containing 100 mL of LB medium and stirred constantly (200 rpm) with magnetic stirrer (Heidolph, Schwabach, Germany) at 29 ± 2 °C for four days. Respiration rates (μL min^−1^), as well as cumulative oxygen consumed (μL) were determined. Cell respiration was measured every 240 min for four days. A sterile medium with cadmium and a *Pseudomonas* – inoculated medium without cadmium were used as growth controls. All experiments were performed in three replicates.

### Protein isolation and identification

#### Protein extract preparation

The metal concentration selected for the proteomic analysis was 0.9 mM of cadmium, as with this sub-lethal concentration, *P. aeruginosa* san ai grows well, developing mechanisms of cadmium resistance (see results). Proteins were isolated from cadmium-amended biomass and control biomass, both in early stationary phase. Biomass was collected after 48 h of growth by centrifugation, frozen and homogenized in two volumes of buffer A (50 mM Tris buffer pH 7.5 supplemented with 0.1 mM of phenylmethylsulfonyl fluoride (PMSF) and 0.5 mM of dithiothreitol) in a glass Teflon homogenizer, at 4 °C. The resulting homogenates were treated at 80 °C for 20 min and ultracentrifuged for 2 h at 100 000 × *g* at 4 °C using Beckman Coulter L5-65, rotor SW 28 (Beckman, Indianopolis, USA).^31^ Three volumes of acetone were added to supernatant and precipitated proteins were collected by centrifugation at 12 000 × *g* for 10 min (Microcentrifuge Force 12, Denver Instruments, New York, USA).^25^ Precipitate was dissolved in a minimal volume of buffer A and applied to Sephadex G-100 column (2.5 × 70 cm) equilibrated in buffer A. Fractions (volumes of 5.5 mL) were collected and absorbance at 280 nm was recorded. Fractions were pooled according to absorbance. Cadmium was determined in pooled fractions. Pooled fractions were lyophilized and kept in the refrigerator prior to use, when solutions of desired concentrations in water were prepared. The protein concentration was determined by the Bradford method.^32^ Two temporary independent column separations were done.

#### Metallothionein preparation

Metallothioneins were isolated using the method of Wang *et al.*^33^ Trypsin digestion was performed overnight as described below and by microwave irradiation as suggested Wang *et al.*^33^

#### In-solution trypsin digestion

Urea (36 mg) and 7.5 μL of 1.5 M Tris buffer at pH 8.8 were added to 100 μL of protein solution, and the mixtures were incubated for 1 h at room temperature. The mixtures were reduced by addition of 2.5 μL of 200 mM tris(2-carboxyethyl)phosphine (TCEP) and incubation for 1 h at 37 °C. Proteins were alkylated by 20 μL of 200 mM iodoacetamide and incubated in the dark for 1 h at room temperature, after which 20 μL of 200 mM of dithiothreitol was added. After incubation at room temperature for 1 h, 800 μL of 25 mM ammonium-bicarbonate and 100 μL methanol (HPLC grade) were added to each tube. Protein was digested with trypsin (Promega Sequencing Grade modified Trypsin dissolved in 25 mM ammonium-bicarbonate) at a ratio of 1 trypsin: 50 protein (2 μg trypsin for 100 μg protein). Digestion was performed overnight at room temperature and concentrated to near dryness in a vacuum concentrator Eppendorf Concentrator 5301 (Eppendorf, Hamburg, Germany). Proteins were desalted by Pierce C18 Spin columns (Pierce Biotechnology, Waltham, Massachusetts, USA) according to manufacturer protocol.

#### Mass spectrometry and HPLC

Peptide digests were analyzed by electrospray ionization in the positive mode on an ion trap instrument Amazon Speed (Bruker, Bremen, Germany), using captive spray source. Two analytical replicates of every sample were done. Peptides were separated by nanoflow HPLC (NanoAdvance, Bruker, Bremen, Germany). UHPLC Nanotrap (100 μm i.d. × 25 mm long) packed with 200 A C_18_ stationary phase (5 μm, C18AQ, Michrom) was used for peptide trapping. Analytical columns (100 μm × 150 mm long) packed with 200 A C_18_ stationary phase (3 μm, C18AQ, Michrom) were coupled to the mass spectrometer (MS).

Peptide mixtures obtained after tryptic digestion were applied to the precolumn at a flow rate of 5 μL min^−1^ in 2% (v/v) acetonitrile with 0.1% (v/v) formic acid. Peptides were eluted by a linear gradient of A (water, 0.1% formic acid) and B (acetonitrile, 0.1% formic acid), as follows: 0 min – A (98%), B (2%); 50 min – A (5%), B (95%); 50–55 min – A (5%), B (95%) with flow rate of 400 nL min^−1^. Ion source conditions were optimized with a calibration solution according to the instrument provider. All MS survey scans were performed from *m*/*z* 400–1400 with enhanced resolution. Data analysis was performed by selection of the five most abundant precursors rejecting singly charged ions.

#### MS data processing, database search and data deposition

Tandem mass spectral data were acquired and processed automatically using Hystar 3.2 and Data Analysis 4.2 software (Bruker, Germany). Deconvoluted .mgf spectra were searched by Easy Prot, a platform for mass spectrometry data processing and protein identification, available at: http://easyprot.unige.ch/.^34^ Search parameters were set as follows: taxonomy-*Pseudomonas* group ID 136841; scoring model – esquire 3000; modifications – methionine oxidation and cysteine alkylated by iodacetamide as variable; enzyme – trypsin, missed cleavages – 2; precursor tolerance – 0.5 Da; peptide: length 6, score 4.0; coverage 15%, series of b; b++; y; y++ were used. To avoid false protein identifications False Discovery Rate (FDR) was kept at l%.

Metallothionein identification was additionally attempted using the Mascot database search tool (version 2.3.02) by querying MS data against a custom metallothionein database. The custom database was assembled out of 2518 protein sequences found to be related to metallothioneins and is listed within the Uniprot knowledge base. Mascot search parameters were set as follows: enzyme –trypsin, missed cleavages – 6, modifications – cysteine carbamidomethylation (global) and methionine oxidation (variable), precursor mass tolerance – 1.2 Da, MS/MS mass tolerance – 0.6 Da, ions with monoisotopic *m*/*z* value and +2 and +3 charge state were tested. Only top hit compounds with significance threshold of *p* < 0.01 were considered.

The mass spectrometry proteomics data have been deposited to the ProteomeXchange Consortium *via* the PRIDE partner repository with the dataset identifier PXD008020. Project name: Cadmium specific proteomic responses of *Pseudomonas aeruginosa* san ai.

## 3. Results and discussion

### Effects of cadmium on *P. aeruginosa* san ai growth

The minimal inhibitory concentration of 7.2 mM of cadmium classifies *P. aeruginosa* san ai in a group of highly resistant *Pseudomonas*.^35–37^ As concentrations which affect metabolic changes in *Pseudomonas* species, but do not induce non-culturability, were reported to be about 15% of MIC,^9,11^ a concentration of 0.9 mM of cadmium was selected for further analysis of the *P. aeruginosa* san ai response to cadmium exposure.

### Extracellular cadmium binding

**Fig. 1:**
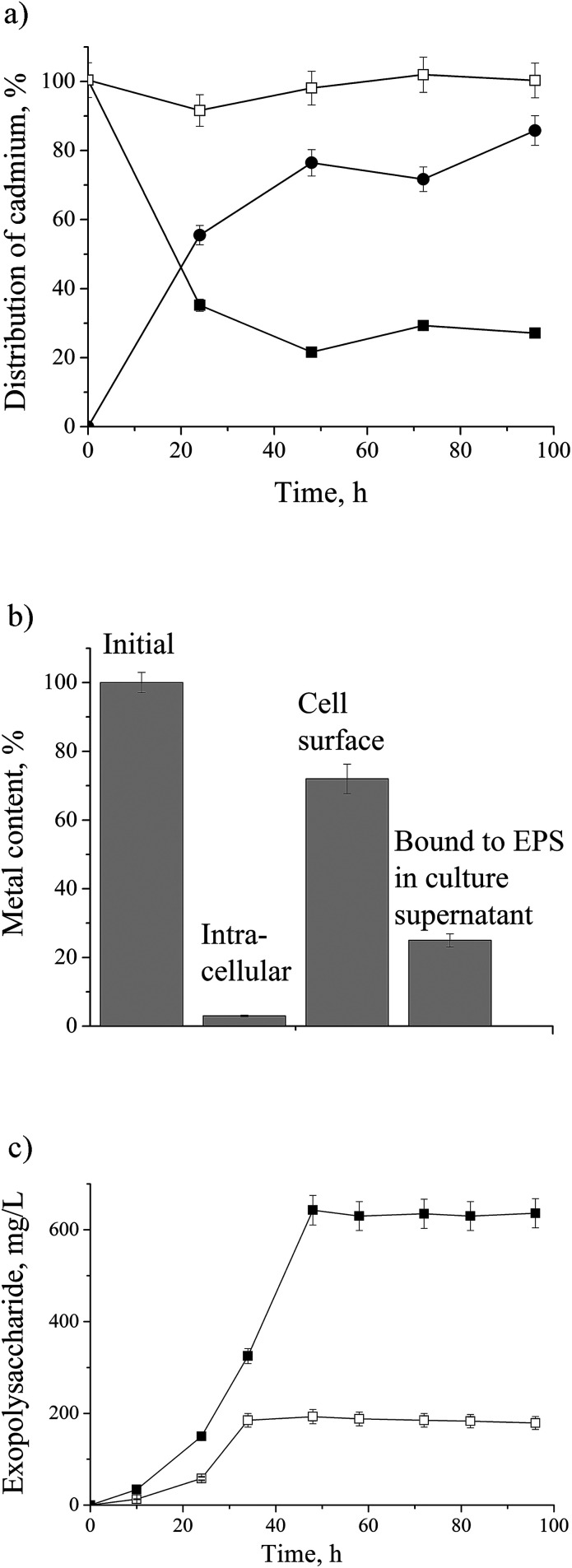
(a) Dynamic of cadmium uptake and distribution in culture broth and biomass of *P. aeruginosa* san ai grown in LB medium with CdCl_2_ (100 mg L^−1^): □-control, ■-cadmium in culture supernatant, ●-cadmium in biomass; (b) intra- and extracellular concentration (bioaccumulation and biosorption) of cadmium; (c) exopolysaccharide concentration in culture broth: □-control, ■-cadmium-amended.

The dynamics of cadmium removal from the culture supernatant and its assimilation by the *P. aeruginosa* san ai are shown in Fig. 1a and b. In the course of exposure to initial concentration of 0.9 mM, 75% of cadmium was accumulated in biomass. *P. aeruginosa* san ai removes 0.307 mM of cadmium per g of biomass after 48 hours, implying a large biosorption potential of leaving biomass. The remaining 25% of cadmium was bound to EPS in the culture supernatant. Extracellular polysaccharides organized around cells or excreted in growth medium greatly contribute to defense mechanisms in *Pseudomonas*.^1,38^ The sorption cadmium uptake by EPS was 0.375 mmol g^−1^. Furthermore, cadmium concentrations found in the cell homogenate was very low (3%) suggesting that main part of cadmium was bound to the cell surface. Concentrations of EPS in the culture supernatant were determined and approximately three times higher production of EPS in cadmium-amended culture was found (Fig. 1c). The alteration of EPS production under the exposure of heavy metal chromium was reported.^38^ The changes in EPS production are related to its protective roles for microbial community such as prevention of dehydration, adhesion, and preserve against environmental stress including antibiotics and toxins, which all together are incorporated in process of biofilm formation. Furthermore, EPS acts as a protective layer by restricting diffusion of some antimicrobial agents into the biofilm by being an ion exchanger.^1^

### Protein response to cadmium

As we aimed to analyze metalloproteins and metabolic pathways related to cadmium exposure in *P. aeruginosa* san ai we used SEC prior to MS analysis. Global analyses of metalloproteins and protein complexes using the gel-free methodology, based on size exclusion chromatography (SEC) has been recently validated.^15^ Proteins were separated in fractions: FI, FII and FIII (ESI, Fig. 1†) and the cadmium content in fractions was determined as (mg L^−1^): 0.144, 0.005 and 0.001, respectively, while no cadmium was found in the control sample (with no added cadmium). Proteins from fractions FI, FII and FIII were analyzed by liquid chromatography coupled with tandem mass spectrometry. Identified proteins in cadmium-amended and control samples were compared and only those proteins which were uniquely found in cadmium-amended samples, but not appeared in control sample were considered as differentially expressed under cadmium exposure.

**Fig. 2:**
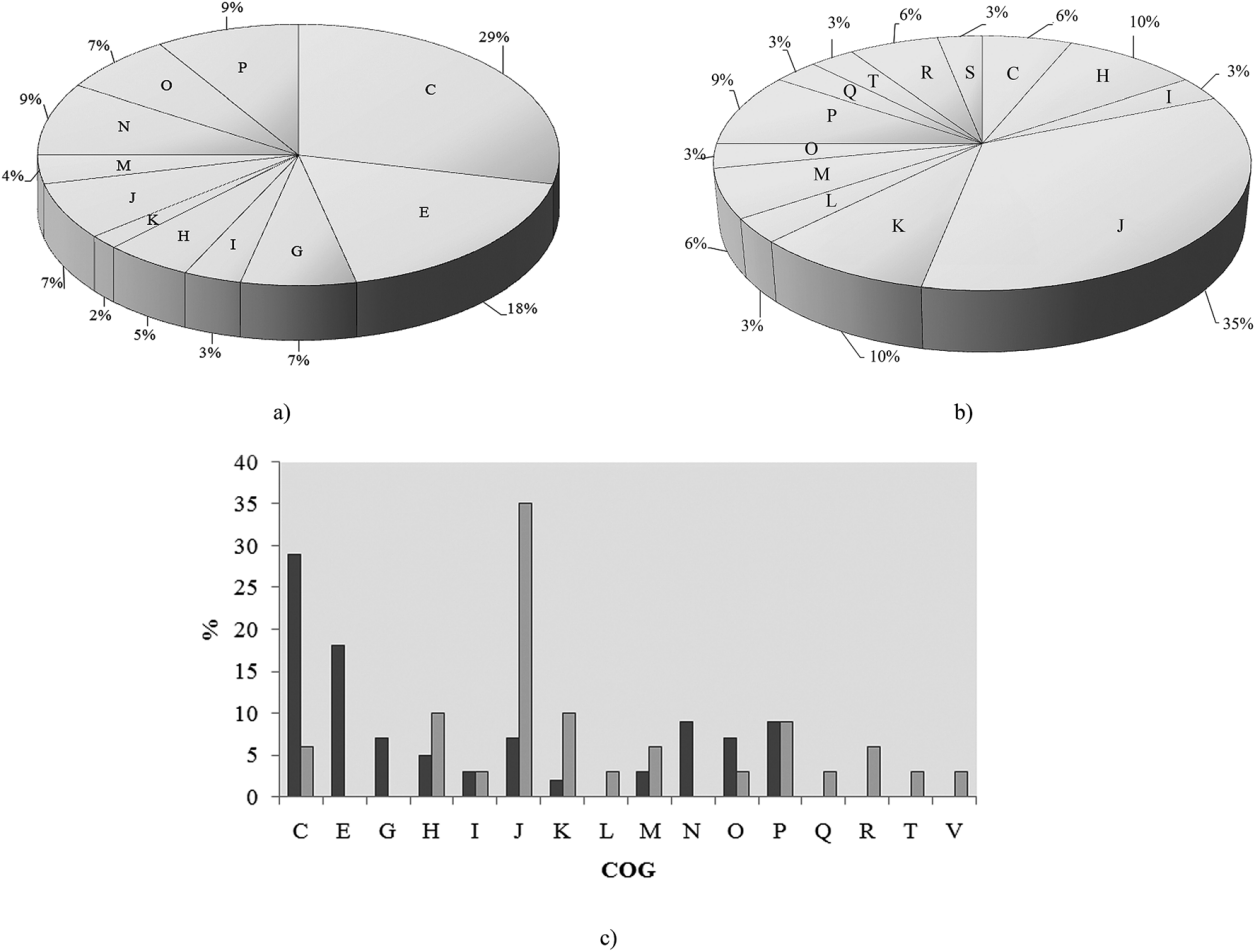
Functional classification of proteins in *P. aeruginosa* san ai. (a) Cd-responsive, (b) control, (c) comparison Cd-responsive *versus* control (dark gray bar – Cd-responsive, light gray – control) according to COG: C – energy production, E – amino acid metabolism, G – carbohydrate metabolism, I – lipid metabolism, J – translation, H – coenzyme metabolism, K – transcription, L – replication and repair, M – cell wall biogenesis, N – cell motility, O – PTM, chaperon function, P – inorganic ion transport, Q – secondary structure, R – general function prediction, T – signal transduction, S – function unknown.

There was a total of 60 proteins differentially expressed in Cd-amended culture which were classified according to COG data base (https://www.ncbi.nlm.nih.gov/COG/) into 11 groups (Fig. 2a) and 32 proteins differentially expressed in control classified into 13 groups (Fig. 2b). Only one protein was found in common in both samples (porin F). Most of the proteins in Cd-amended culture functionally belong to: energy production and conservation, amino acids metabolism, posttranslational modifications and cell motility, while in control prevailed proteins of translation and transcription. The comparison of protein functions in Cd-amended and control culture reveals quite different patterns (Fig. 2c). List of proteins with identified peptide sequences in cadmium-amended and control samples are shown in ESI (Tables 1, 2 and 3†).

### Metalloproteins

Cadmium resistance of *P. aeruginosa* is accompanied by up-regulation of metalloproteins uniquely found in cadmium-amended culture, most of which are located in the outer membrane or periplasm – the first line of the cell defense. The crystal ionic radius of Cd^2+^ (in pm) of 109, being close to the radii of Hg^2+^ (116) and Ca^2+^ (114),^39^ significantly determines the specificity of the cadmium response in bacterial cells. We have identified 16 metalloproteins that represent almost 30% of all over-expressed proteins (ESI, Table 1†). The greatest part of metalloproteins observed in our study belongs to two COG categories – inorganic ion transport and metabolism (*P*) and energy production and conversion (*C*).

**Fig. 3:**
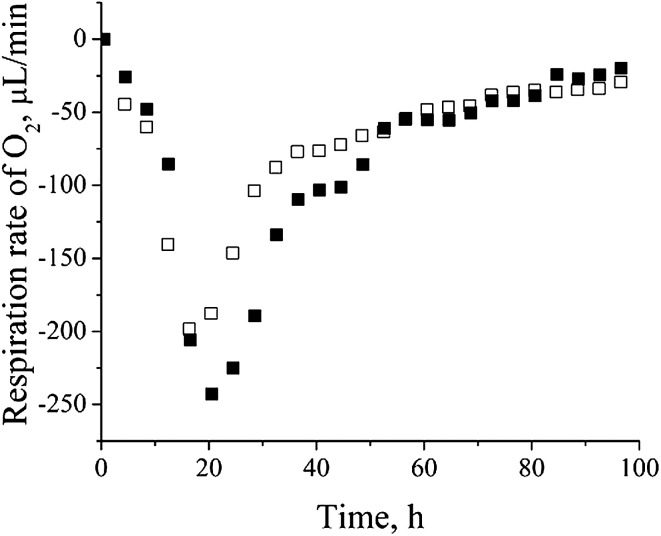
Oxygen respiration rate of control *versus* cadmium-amended culture of *P. aeruginosa* san ai: □ – control, ■ – cadmium-amended.

*P. aeruginosa* san ai is a denitrificator and as such it possesses dissimilatory nitrate respiration system^19^ (GenBank accession number JMKR00000000) composed of metalloproteins: azurin (AZUR) – copper containing, cytochrome C551 (CY551) – iron containing, nitrite reductase (NIRS) – iron containing, and nitrous-oxide reductase (NOSZ) – copper and calcium containing.^40^ We believe that cadmium replaced the mentioned metals in denitrification proteins. Copper substitution by cadmium in the blue copper redox metalloprotein azurin (AZUR) was already reported and it is based on similar metal (cadmium and copper) coordination and binding sites containing cysteine and histidine.^41^ Also, cadmium competition with iron in heme and Fe–S proteins was already observed.^42,43^ Exchange of essential metals (Fe, Cu, Ca, *etc.*) with cadmium in metalloproteins with non-redox function will probably lead to a change, but not to a lost of their metabolic activity, while replacing redox metal with Cd will certainly lead to the complete inactivation of a particular protein. However, in conditions of overproduction of metalloproteins, and at relatively low concentration of cadmium, it is possible that part of the metalloproteins completely saved their metal ions and thus the function. Low sub-lethal concentrations of cadmium, influences fine-tuning of metabolic and energy system in process of adaptation. On the other hand, high concentrations of cadmium cause a massive change in the total metabolic picture (data not shown). In order to confirm the thesis that denitrification system is not active we determined the content of nitrate and nitrite in culture filtrate and respiration rate of culture. Very low content of 0.125 mM nitrate and 0.005 mM nitrite was far less than concentrations reported as necessary for denitrification.^44^ Oxygen respiration rate (Fig. 3) clearly showed increased oxygen consumption in a cadmium-amended culture implying that *P. aeruginosa* san ai did not activate its machinery for denitrification in given conditions, thus these proteins seemingly serve as a metal ion depot. Proteins of denitrification cascade which is not active, obviously, can protect microbial cells against heavy metals. In accordance with our observation Gui *et al.*^45^ found that aerobic denitrification was inhibited with heavy metals, including cadmium. Based on the similarity of ionic radii, Cd^2+^ probably replaced mercury in mercuric transport protein (MERP). Similar to our finding, Jain *et al.*^46^ reported the expression of mercuric resistance protein (MERD) in *P. monteilii* in the presence of cadmium.

**Fig. 4:**
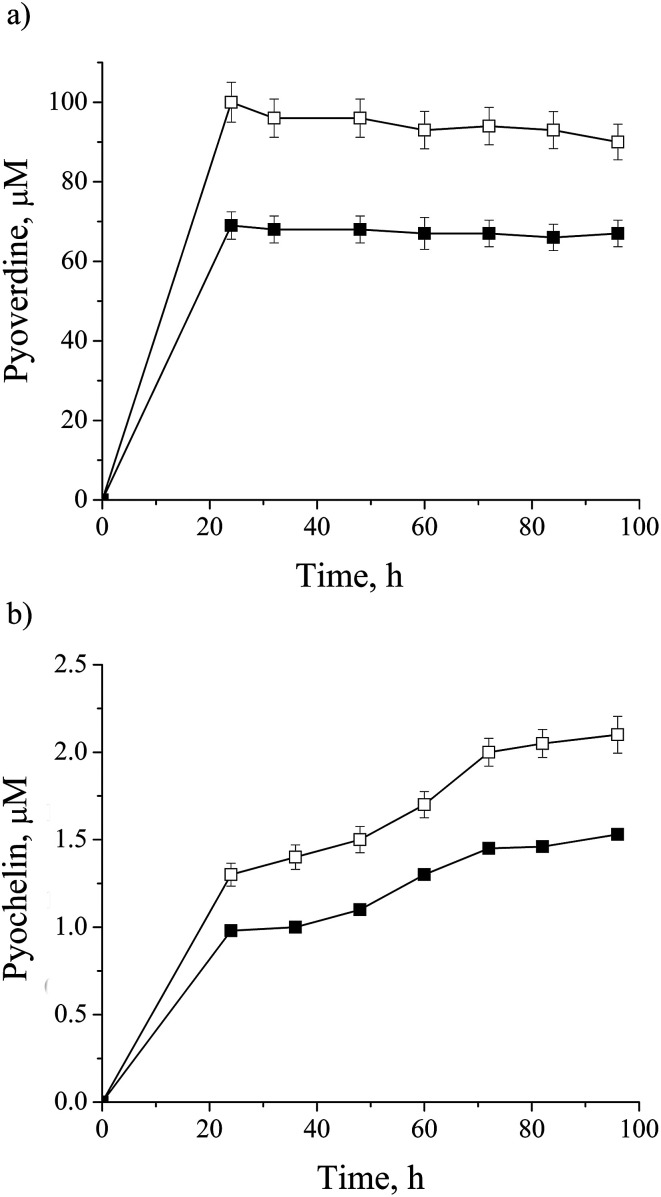
Dynamic of siderophore production by *P. aeruginosa* san ai: (a) pyoverdine, (b) pyocheline in: □ – control, ■ – cadmium-amended. Each data point is the mean of three different parallel cultures.

Most of the metalloproteins found to be impacted by cadmium are iron containing: NIRS, CY551, catalase (CATA), superoxide dismutase (SODF), bacterioferritin (BFR), Fe(3+)-pyochelin receptor (FPTA) and molybdenum cofactor biosynthesis protein (MOAA1) as a Fe–S cluster protein (ESI, Table 1†). As already mentioned, iron could be substituted by cadmium.^42,43^ On the other side, iron concentration is essential for cell homeostasis, so some microorganisms synthesize siderophores to control iron bioavailability. *P. aeruginosa* san ai produces pyoverdine, a highly iron-specific siderophore, and pyochelin, a siderophore with a broad specificity,^14,47^ and they were both monitored in the culture supernatant and depicted in Fig. 4a and b, respectively. We observed the down-regulation of both siderophores which was in agreement with results reported by Teitzel *et al.*^14^ and Poirier *et al.*^12^ In addition we identified ferric uptake regulation protein (FUR) responsible for negative regulation of siderophore biosynthetic process and an iron storage protein BFR, similar to Poirier *et al.*^11^ These results clearly show the effect of cadmium on the iron homeostasis.

Expected metallothioneins were not found in this investigation. Possible reasons for their absence include their time-dependent production and low concentration, along with the problematic digestion and preparation procedures for MS analysis, which have been already reported.^9^

### Cell energy metabolism

**Fig. 5:**
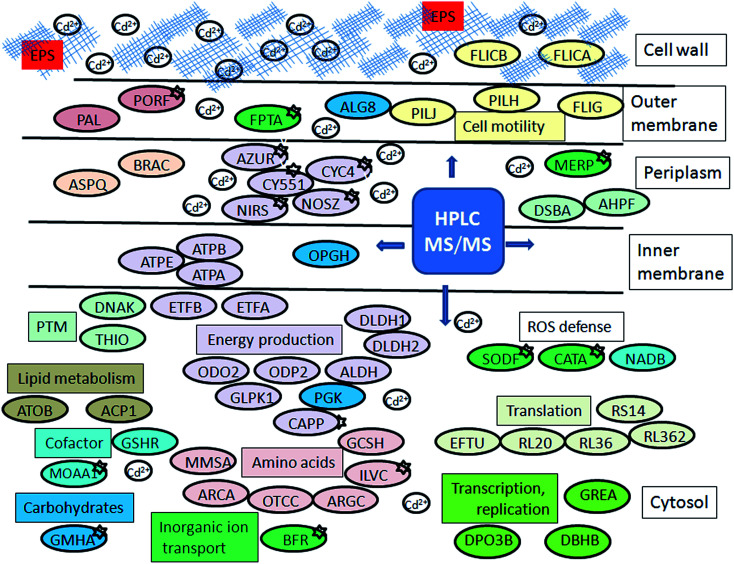
Schematic model of *P. aeruginosa* san ai response to cadmium. Protein abbreviations are the same as in Tables 1 and 2. Proteins which belong to the same COG are labeled by the same colour.

Up-regulation of protein biosynthesis – ESI† (Table 2) caused by cadmium in *P. aeruginosa* san ai expectedly had an increased energy requirement.^8^ Up-regulation of electron transfer flavoprotein, ETFA/B involved in electron transport to the main respiratory chain suggested enhanced respiration, whereas up-regulation of ATP synthase implied an increased production and consumption of ATP (Fig. 5). Over-expression of the dihydrolipoyl dehydrogenase component of the α-ketoacid dehydrogenases complex, together with components of α-ketoglutarate dehydrogenase (2-oxoglutarate dehydrogenase) and pyruvate dehydrogenase complexes, produce NADH and provide electrons for the respiratory chain. So, the activity of these enzymes is of crucial importance in the stimulation of the respiratory chain and consequently in ATP supply under conditions of an increased ATP demand in cadmium exposed cells. In addition, produced keto acids might contribute cadmium chelation, preventing heavy metal stress. Cadmium-amended culture in exponential phase showed oxygen consumption of 7.14 μmol × min^−1^ g^−1^*versus* 5.19 μmol × min^−1^ g^−1^ in control. An increased O_2_ consumption of 37% in exponential phase and 30% in early stationary phase well document a more intense respiration in cadmium-amended culture than in control (Fig. 3).

### Amino acid metabolism

As Table 2 shows, an intensive amino acid metabolism took place when *P. aeruginosa* san ai was exposed to cadmium. Arginine, lysine, glycine, histidine, asparagine, and valine metabolism, as well as the transport of branched amino acids were affected by cadmium (Table 2, Fig. 5 and ESI,† Table 2). Increased amino acid metabolism is certainly connected to protein overexpression. In addition, up regulation of the glycine cleavage system, GCSH which is highly sensitive to alterations in the oxidation–reduction state of the respiratory chain (*e.g.* oxidizing conditions stimulate while reducing conditions strongly inhibit this process), was observed.^48^ Arginine deiminase and catabolic ornithine carbamoyltransferase, enzymes of arginine degradation by the arginine deiminase (ADI) pathway were identified. ADI is a non-redox, ATP producing process which can be induced under various stress conditions.^49,50^ Actually, the amino acid catabolism is the main effect of cadmium and can be considered in relation to: improved energy production, biosynthesis of basic building blocks for other structural molecules, or particular amino acid like cysteine for cadmium sequestering.

**Table 1:** Metalloproteins from *Pseudomonas aeruginosa* san ai up-regulated in the presence of 0.9 mM cadmium, classified according to COG^a^

Identified protein	Entry name^b^	Easy Prot score	Number of matched peptides	Sequence coverage, %	Metal (according to Uniprot)	Molecular mass kDa	COG^a^	SEC^c^ fraction
Azurin	AZUR_PSEAE	116	4	42	Cu	13.9	C	I
Nitrous-oxide reductase, [chain 0]	NOSZ_PSEAE	152	5	18	Ca, Cu	65.8	C	I
Nitrite reductase/cytochrome cd1	NIRS_PSEAE	58	4	9	Fe	62.6	C	I
Cytochrome c-551	CY551_PSEAE	33	2	27	Fe	10.9	C	I
Cytochrome c4 [chain 0]	CYC4_PSEAE	23	2	7	Fe	18.6	C	I
Phosphoenolpyruvate carboxylase (PEPCase)	CAPP_PSEAE	20	2	4	Mg	97.8	C	I
Malate synthase G	MASZ_PSEAE	79	5	10	Mg	78.5	C	I
Bacterioferritin	BFR_PSEAE	34	2	12	Fe	17.9	P	I
Fe(3+)-pyochelin receptor	FPTA_PSEAE	68	4	5	Fe	76	P	I
Catalase	CATA_PSEAE	73	4	8	Fe	55.5	P	I
Superoxide dismutase [Fe]	SODF_PSEAE	14	1	6	Fe	21.3	P	I
Mercuric transport protein periplasmic component	MERP_PSEAI	77	3	55	Hg	9.3	P	II
Phosphoheptose isomerase	GMHA_PSEAE	55	3	19	Zn	21.4	G	I
Molybdenum cofactor biosynthesis protein A 1	MOAA1_PSEAE	36	4	9	Fe, 4Fe–4S	36.6	H	I
Ketol-acid reductoisomerase (NADP(+))	ILVC_PSEA8	101	4	15	Mg	36.4	H	I
Ferric uptake regulation protein	FUR_PSEAE	92	3	3	Fe, Zn	15	P	II
Outer membrane porin F	PORF_PSEAE	111	6	28	Ca	37.6	M	I

a According to https://www.ncbi.nlm.nih.gov/COG/.

b Matching microorganism sources are: PSEAE – *P. aeruginosa* ATCC 15692/PAO1, PSEAI – *P. aeruginosa*, and PSEA8 – *P. aeruginosa LESB58*.

c Size exclusion chromatography fraction.

**Table 2:** *Pseudomonas aeruginosa* san ai non-metalloproteins up-regulated in the presence of 0.9 mM cadmium, classified according to COG^a^

Identified protein	Entry name^b^	Easy Prot score	Peptide matched	Coverage (%)	Molecular mass (kDa)	COG^a^	SEC^c^ fraction
Electron transfer flavoprotein subunit, β	ETFB_PSEAE	101	4	23	26.3	C	I
Electron transfer flavoprotein subunit, α	ETFA_PSEAE	92	5	16	31.4	C	I
ATP synthase subunit alpha	ATPA_PSEA8	75	4	10	55.3	C	I
ATP synthase subunit beta	ATPB_PSEAE	39	3	7	49.5	C	I
ATP synthase epsilon chain	ATPE_PSEAE	35	2	16	14.7	C	I
Dihydrolipoyl dehydrogenase1	DLDH1_PSEAE	160	7	25	48.6	C	I
Aldehyde dehydrogenase	ALDH_PSEOL	34	4	9	52.8	C	I
Dihydrolipoyllysine-residue succinyltransferase component of 2-oxoglutarate dehydrogenase complex	ODO2_PSEAE	50	3	10	42.9	C	I
Glycerol kinase	GLPK1_PSEAE	190	9	22	55.9	C	I
Dihydrolipoyllysine-residue acetyltransferase component of pyruvate dehydrogenase complex	ODP2_PSEAE	32	3	9	56.7	C	I
Arginine deiminase	ARCA_PSEAE	27	2	9	46.4	E	I
Ornithine carbamoyltransferase, catabolic	OTCC_PSEAE	68	4	13	38	E	I
*N*-acetyl-gamma-glutamyl-phosphate reductase	ARGC_PSEAE	30	2	8	36.7	E	I
Glycine cleavage system H protein 1	GCSH1_PSEAE	30	2	16	13.7	E	II
Glycine cleavage system H protein 2	GCSH2_PSEAE	28	2	15	13.6	E	II
Glutaminase-asparaginase	ASPQ_PSEAE	32	2	13	38.6	E	II
Leucine-, isoleucine-, valine-, threonine- and alanine-binding protein	BRAC_PSEAE	58	3	23	39.7	E	I
Methylmalonate-semialdehyde dehydrogenase	MMSA_PSEAE	80	4	12	53.5	E	I
Phosphoglycerate kinase	PGK_PSEAE	64	4	16	40.4	G	II
Glycosyltransferase alg8	ALG8_PSEAE	19	2	5	56.5	G	I
Glucans biosynthesis glucosyltransferase H	OPGH_PSEA7	32	4	4	96	G	I
Acetyl-CoA acetyltransferase	ATOB_PSEAE	20	2	3	40.3	I	II
Acyl carrier protein 1	ACP1_PSEAE	50	2	19	8.7	I	III
l-Aspartate oxidase	NADB_PSEAE	17	2	4	60	H	I
Glutathione reductase	GSHR_PSEAE	22	3	5	49	H	I
Transcription elongation factor GreA	GREA_PSEAE	77	3	25	17.1	K	I
DNA-binding protein HU-beta	DBHB_PSEAE	44	2	32	9	K	III
DNA polymerase III subunit beta	DPO3B_PSEAE	51	3	8	40.6	L	I
Elongation factor Tu	EFTU_PSEAB	38	2	5	43.3	J	III
50S ribosomal proteins L36 2	RL362_PSEAB	42	3	86	5.9	J	III
50S ribosomal proteins L 20	RL20_PSEAE	22	2	13	13.3	J	III
30S ribosomal proteins S14	RS14_PSEAE	21	2	21	11.5	J	III
50S ribosomal proteins L36	RL36_PSEAE	24	2	58	4.4	J	III
Peptidoglycan-associated lipoprotein	PAL_PSEAE	68	3	31	15.8	M	II
Protein PilJ	PILJ_PSEAE	26	2	4	72.5	N	I
Protein PilH	PILH_PSEAE	72	3	23	13.2	N	II
A-type flagellin [chain 0]	FLICA_PSEAI	82	4	13	39.9	N	II
B-type flagellin	FLICB_PSEAE	30	2	5	49	N	I
Flagellar motor switch protein FliG	FLIG_PSEAE	28	3	9	37	N	II
Thiol:disulfide interchange protein DsbA	DSBA_PSEAB	35	2	12	23.3	O	II
Thioredoxin	THIO_PSEAE	86	4	43	11.8	O	II
Chaperone protein DnaK	DNAK_PSEAE	123	6	11	68.3	O	I
Alkyl hydroperoxide reductase subunit/thioredoxin peroxidase	AHPF_PSEAE	22	3	12	20.5	O	II

a According to https://www.ncbi.nlm.nih.gov/COG/.

b Matching microorganism sources are: PSEAE – *P. aeruginosa* ATCC 15692/PAO1, PSEAB – *P.aeruginosa*, UCBPP-PA14, PSEAI – *P. aeruginosa*, PSEA7 – *P. aeruginosa* PA7, PSEA8 – *P. aeruginosa* PA8 and PSEOL – *P. oleovorans*.

c Size exclusion chromatography fraction.

### Protein biosynthesis and posttranslational modifications

The excess of cadmium caused up-regulation of protein biosynthesis in *P. aeruginosa* san ai (Table 2 and ESI, Table 2†). The transcription elongation factor, GREA regulates transcription, while the factor EFTU and several 30S and 50S ribosomal proteins contribute to protein translation. Damaged proteins were repaired by chaperone DNAK. Posttranslational modifications are mainly related to protein redox processing based on the thiol group from cysteine. The thiol-containing molecules can serve to chelate heavy metal and thus prevent binding of cadmium to functionally important proteins. Cadmium interaction with biomolecules, in particular its reactivity toward the thiol group is based on a very low solubility product of CdS, (*K*_sp_ = 1 × 10^−29^). Once transported into the cytoplasm, cadmium cation binds to free thiol groups disrupting protein structure and function.^10,14^ As redox exchange of cysteine residue cannot exist in isolation several redox-active proteins were identified as up-regulated. Revealed redox cascade, based on the thiol-disulfide exchange, with oxidation–reduction potentials ranging from −279 mV (thioredoxin, THIO), −243 mV (glutathione reductase, GSHR) to −124 mV (thiol: disulfide interchange protein, DSBA),^51^ clearly implies that cadmium affects peptides and proteins containing sulfhydryl groups. Delalande *et al.*^52^ confirmed that the formation of Cd(GS)_2_ under physiological conditions is spontaneous. On the other side, the ratio GSH/GSSG is kinetically controlled by GSH-utilizing enzymes,^53^ from which thioredoxin and glutathione reductase were identified in this study.

It has been recently proven that the mechanism of cadmium toxicity involves the production of reactive oxygen species (ROS).^54^ As cadmium is a redox inactive metal ion, production of free radicals and ROS probably occurs through an indirect mechanism.^55^ To neutralize ROS, apart from metalloproteins superoxide dismutase SODF and catalase CATA, alkyl hydroperoxide reductase AHPF was up-regulated in the current study (Table 2, Fig. 5). AHPF is a kinetically more potent H_2_O_2_ scavenger than catalase, and it is probably the primary scavenger of endogenous H_2_O_2_.^56^ To combat oxidative stress flavoenzyme l-aspartate oxidase (NADB) was up-regulated in *P. aeruginosa* san ai when exposed to cadmium (Table 1). NADB catalyzes the first step in the biosynthesis of NADP^+^ and NAD.^57^

### Carbohydrate metabolism

As 2-oxoglutarate and pyruvate can be formed from several amino acids,^49^ overexpression of 2-oxoglutarate dehydrogenase and pyruvate dehydrogenase could be considered as a result of emphasized amino acid metabolism. 2-Oxoglutarate dehydrogenase involved in Krebs cycle and pyruvate dehydrogenase at Krebs cycle entrance are two main cellular multienzyme complexes, which enable effective energy supply. In the same time, pyruvate is an carbohydrate precursor which can be converted into glucose which is further involved in biosynthesis of lipopolysaccharide and alginate. Indeed, *P. aeruginosa* san ai exposed to cadmium showed up-regulation of: peptidoglycan-associated lipoprotein-PAL, phospho heptose isomerase GMHA, responsible for lipopolysaccharide biosynthesis, and glycosyltransferase ALG8, involved in alginate biosynthesis (Fig. 5). Over-expression of these proteins suggested an increased biosynthesis of outer membrane polymers according to previous observations.^8^ These polymers on the cell surface and EPS released to culture supernatant were bound to the main part of cadmium, as shown in Fig. 1c. Exopolysaccharide identified as alginate^18^ is a linear polymer composed of d-mannuronic acid and l-guluronic acid, and as such greatly contributed to heavy metal binding, reducing cadmium interaction with the microbial cell.

### Cell motility

Proteins PilH and PilJ as well A- and B-type flagellin, were found up-regulated in this study (Table 2, Fig. 5 and ESI, Table 2†). Cell surface structures composed from these proteins, pilli and flagella, together with EPS and lipopolysaccharides (LPS), are involved in the early stages of *P. aeruginosa* biofilm formation.^58,59^ More precisely, pili regulate twitching motility, while flagella has important role in microbial adhesion.^60–63^ and the use of both, enables *P. aeruginosa* cells to colonize and rapidly explore microenvironments.^60,61^ However, Poirier *et al.*^11^ reported a down-regulation of flagellin synthesis and a decreased mobility of *P. fluorescens* BA3SM1 under the exposure to cadmium, zinc and copper, explaining this phenomenon as a bacterial response to economize energy. In addition to motility proteins, an increased amount of EPS alginate and up-regulation of LPS biosynthesis (protein GMHA) were observed – these two molecules are of crucial importance for biofilm formation. Previously in the discussion (section Extracellular cadmium binding) it was notified important role of EPS as protective layer against toxic effect of cadmium acting as an ion exchanger.^1^ As negatively charged EPS efficiently bound cadmium in supernatant in our study, according to observation of van Hullebush *et al.*,^64^ it is obvious that biofilm formation induced by heavy metal presence^1^ is one more element of the complex metal resistance strategy in *P. aeruginosa* san ai exposed to cadmium, as it was observed in *P. putida* KT2440.^9^

## 4. Conclusion

*Pseudomonas aeruginosa* san ai resists a high concentration of up to 7.2 mM of cadmium and as such is ecologically relevant strain with the potential to be applied in bioremediation of heavy metal polluted environments. Under exposure to cadmium, *P. aeruginosa* san ai exhibits two main mechanisms: stress and defense response. *P. aeruginosa* san ai evolved several defense strategies at both intracellular and extracellular levels: (1) extracellular binding of cadmium to exopolysaccharide released in culture broth, (2) binding of cadmium to the cell surface, (3) elevated production of metalloproteins, particularly denitrification proteins, (4) metabolic adaptation to increased concentration of cadmium inside the cell.

Biomass of *P. aeruginosa* san ai exposed to sub-lethal concentration of cadmium (0.9 mM) adsorbed 75% of added cadmium, while remaining 25% in culture supernatant was adsorbed by EPS, implying a large biosorption potential of leaving biomass and EPS. Due to EPS negatively charged chemical groups it has a protective role preventing cadmium entry to the cells. In addition, increased biosynthesis of exopolysaccharide, lipopolysaccharide, pilli and flagellar proteins unambiguously indicates biofilm formation as additional strategy to combat heavy metal outside the cell.

Inner cell response on the protein level was investigated by global proteomics approach based on protein prefractionation by size exclusion chromatography which was used to preserve native forms of metalloproteins and protein complexes, followed by liquid chromatography and tandem mass spectrometry coupled with bioinformatics to identify proteins. Almost a third of the total number of 60 differentially expressed proteins in the presence of cadmium were metalloproteins. The fraction in which the metalloproteins prevail has the highest measured cadmium content indicating metal ion replacement in these proteins. Since *P. aeruginosa* san ai tolerates very high concentrations of cadmium (up to 7 mM), its exposure to 0.9 mM likely did not cause significant loss of biological activity of the cadmium supstituted proteins, presumably because the level of residual essential metal in the proteins remained sufficient for their function.

As in the cytoplasm cadmium cation binds to free thiol groups from proteins and peptides, identified proteins from redox cascade: thioredoxin, glutathione reductase, and thiol: disulfide interchange protein, clearly implies that cadmium affects peptides and proteins containing sulfhydryl groups, as well that *P. aeruginosa* san ai has a potent mechanisms to maintain cellular –SH redox homeostasis. Replacement of iron by cadmium in metalloproteins alters iron availability, so the down-regulation of siderophores and the over expression of ferric uptake regulation protein responsible for negative regulation of siderophore biosynthesis clearly indicated the effect of cadmium on the iron homeostasis in *P. aeruginosa* san ai. Accumulation of iron cation which triggers Fenton's reaction elevates expression of superoxide dismutase, catalase, and alkyl hydroperoxide reductase to suppress accumulation of ROS.

Our results point to overexpression of the mercury ion transporter, MERP. As cadmium and mercury (2+) cations physically differ relatively slightly, it seems possible that MERP might be a good candidate for cadmium transport in *P. aeruginosa* san ai.

Enhanced activities of NAD – dependent dehydrogenases and glycine cleavage system firmly affected respiration, so an enhanced consumption of oxygen in cadmium-amended culture, was measured implying complex cadmium effects on the environment. The present study indicated that the presence of cadmium as a non-essential, non-redox metal induced in *P. aeruginosa* san ai elevated synthesis of carbohydrates and proteins as the major players in the process of cadmium elimination for *P. aeruginosa* san ai.

Cadmium resistance of *P. aeruginosa* san ai is a branched network of powerful extracellular and intracellular adaptation mechanisms, whose harmonic functioning apparently showed a significant potential of the bacteria for its application in environmental heavy metal pollution and co-pollution. Regarding to the observed response the isolate cadmium removal will involve biofilm formation, sequestration by extracellular polymers, and the metal up take by biomass. A deeper understanding of *P. aeruginosa* san mechanisms involved in cadmium resistance and optimal conditions for efficient biosorption are crucial for its successful application in bioremediation.

## Conflicts of interest

There are no conflicts to declare.

## Supplementary Material

RA-008-C8RA00371H-s001
