# Supplementary material for: Cadmium specific proteomic responses of a highly resistant Pseudomonas aeruginosa san ai
Source: RSC Adv. 2018 Mar 16;8(19):10549–60. doi: 10.1039/c8ra00371h (PMC9078880; doi:10.1039/c8ra00371h)
Supplement: RA-008-C8RA00371H-s001 [file RA-008-C8RA00371H-s001.pdf]

Electronic Supplementary Material (ESI) for RSC Advances.
This journal is © The Royal Society of Chemistry 2018

Electronic Supplementary Information for RSC Advances

Cadmium specific proteomic responses of a highly resistant *Pseudomonas aeruginosa* san ai

Table 1. Metalloproteins differentially expressed in the presence of 0.9 mM cadmium in *Pseudomonas aeruginosa*, classified according to COG[a] and identified on the basis of at least two matched peptides and FDR 1%.

Identified protein (strain, species)	Entry name	Easy Prot score	Number of matched peptides	Sequence coverage, %	Peptide sequence	Metal (according to Uniprot)	Molecular mass kDa
**C-** Energy production and conversion							
Azurin (*P. aeruginosa* ATCC 15692/PAO1)	AZUR_PSEAE	116	4	42	K|DSVTFDVSK<br>K|LIGSGEKDSVTFDVSK<br>K|QFTVNLSHPGNLPK<br>K|LKEGEQYMFFCTFPGHSALMK	Cu	13.9
Nitrous-oxide reductase, [CHAIN 0] (*P. aeruginosa* ATCC 15692/PAO1)	NOSZ_PSEAE	152	5	18	R|AEKDGIDLMK<br>R|GNAYTTLFIDSQLVK<br>R|IPVFNVDSATGWGLTNESK<br>R|LGDLFAGK<br>K|LSPTCTMIAIER<br>R|VLGVPSMR<br>K|WNLADAVR<br>K|YLFINDK	Ca, Cu	65.8
Nitrite reductase/ Cytochrome cd1 (*P. aeruginosa* ATCC 15692/PAO1)	NIRS_PSEAE	58	4	9	R|ALRFSAGSWR<br>K|ARLFDWLKR	Fe	62.6
Cytochrome c-551 (*P. aeruginosa* ATCC	CY551_PSEAE	33	2	27	K|FAGQAGAEAELAQR<br>K|SKPCGACHSVQAK	Fe	10.9

15692/PAO1)							
Cytochrome c4 [CHAIN 0] (*P. aeruginosa* ATCC 15692/PAO1)	CYC4_PSEAE	23	2	7	M|ALTVNTNIASLNTQR<br>K|EVAAQQAELTR	Fe	18.6
Phosphoenolpyruvate carboxylase (PEPCase) (*P. aeruginosa* ATCC 15692/PAO1)	CAPP_PSEAE	20	2	4	R|AQEKLVEICR<br>R|EVLLLAR	Mg	97.8
Malate synthase G (*P. aeruginosa* ATCC 15692/PAO1)	MASZ_PSEAE	79	5	10	R|AFLDEAAPLESGSHVDATSYSVK<br>R|TGDEIHTSMEAGAVVR<br>R|TGDEIHTSMEAGAVVR<br>R|VEDVLGLPR<br>K|VPDINDVGLMEDR<br>R|VVFINTGFLDR	Mg	78.5
**P**- Inorganic ion transport and metabolism							
Bacterioferritin (*P. aeruginosa* ATCC15692/PAO1)	BFR_PSEAE	34	2	12	R|ILLLEGTPR<br>R|MGLENYLQSQIK	Fe	17.9
Fe(3+)-pyochelin receptor (*P. aeruginosa* ATCC15692/PAO1)	FPTA_PSEAE	68	4	5	R|AEVDVGGPLSASGNVR<br>R|EFAASTTLSAGR<br>K|LAEPLTLVVGGR<br>R|SLDANLNGPVR	Fe	76
Catalase (*P. aeruginosa* ATCC 15692/PAO1)	CATA_PSEAE	73	4	8	R|LTTAAGAPVVDNQNVQTAGPR<br>R|GVADALGLK<br>K|MYVQIMPEK<br>R|YNPFDLTK	Fe	55.5

Ferric uptake regulation protein	FUR_PSEAE	92	3	30	K|ALMEAGEDVGTVYR<br>R|VLTQFEAAGLVVR<br>K|ILQMLDSAEQR	Fe, Zn	15.2
Superoxide dismutase [Fe] (*P. aeruginosa* ATCC 15692/PAO1)	SODF_PSEAE	14	1	6	M|AFELPPLPYEK	Fe	21.3
Mercuric transport protein periplasmic component (*P. aeruginosa*)	MERP_PSEAI	77	3	55	R|ATQTVTLSVPGMTCSACPITVKK<br>R|QAVVTFDDAK|T<br>K|VDVTFETR|Q	Hg	9.3
**G-** Carbohydrate transport and metabolism							
Phosphoheptose isomerase (*P. aeruginosa* ATCC 15692/PAO1)	GMHA_PSEAE	55	3	19	R|DGGGMASLLLPEDVEIR<br>R|EMLVVALTGR<br>R|QLFQASIETK	Zn	21.4
**H**- Coenzyme transport and metabolism							
Molybdenum cofactor biosynthesis protein A 1 (*P. aeruginosa* ATCC 15692/PAO1)	MOAA1_PSEAE	36	4	9	R|CDFRCVYCMAEDMRFLPR<br>R|CVYCMAEDMR<br>R|CVYCMAEDMRFLPR	Fe, 4Fe-4S	36.6
Ketol-acid reductoisomerase (NADP(+)) (*P. aeruginosa*, strain LESB58)	ILVC_PSEA8	101	4	15	R|ADLDVIMIAPK<br>K|DSGVDVTVGLR<br>R|NNAAHPIEQIGEK<br>K|NVALSYACGVGGGR	Mg	36.4
**M-** Cell wall/membrane biogenesis							
Outer membrane porin F (*P. aeruginosa* ATCC15692/PAO1)	PORF_PSEAE	111	6	28	R|DVLVNEYGVEGGR<br>R|RVEAEVEAEAK<br>R|YFTDSVRNMK<br>R|VQLDVKFDFDKSK	Ca	37.6

				R|VNAVGYGESRPVADNA TAEGR K|QYPSTSTTVEGHTDSVGTDA YNQK			

[a]according to https://www.ncbi.nlm.nih.gov/COG/

Table 2. Proteins differentially expressed in the presence of 0.9 mM cadmium in *Pseudomonas aeruginosa*, classified according to COG[a] and identified on the basis of at least two matched peptides and FDR 1%.

COG category/ Identified protein (strain, species)	Entry name	Easy Prot score	Number of matched peptides	Peptide sequence	Coverage %	Fraction[b]	Molecular mass, kDa
**C-** Energy production and conversion							
Electron transfer flavoprotein subunit beta (*P. aeruginosa* ATCC 15692/PAO1)	ETFB_PSEAE	101	4	K|AVVDKEQPQLVILGK K|GVATEIVAVSVGPTAAQREQ LR K|MSMNPFCEIAVEEAVR K|VEVAGDKVNVTR	23	I	26.3
Electron transfer flavoprotein subunit alpha (*P. aeruginosa* ATCC 15692/PAO1)	ETFA_PSEAE	92	5	R|AAVDAGFVPNDMQVGQTG K K|NYSHVLAPATTNGK K|RPIYAGNAIATVQSSAAVK K|SAFVGEELAK K|VLVADNAAYAHQLPENVA PLIAELGK	16	I	31.4
ATP synthase subunit alpha (*P. aeruginosa,* strain *LESB58)*	ATPA_PSEA8	75	4	R|GQRELIIGDR R|ILEVPVGPELLGR R|NEGTIVSVSDGIVR	10	I	55.3

				R|VVDALGNPIDGK			
ATP synthase subunit beta (*P. aeruginosa* ATCC 15692/PAO1)	ATPB_PSEAE	39	3	R|NDSSLIAIR<br>R|RLEAAGYR<br>R|YATNSETAGFFR	7	I	49.5
ATP synthase epsilon chain (*P. aeruginosa* ATCC 15692/PAO1)	ATPE_PSEAE	35	2	R|AGDLDEAAAQEALK<br>K|VLADTVVR	16	I	14.7
Dihydrolipoyl dehydrogenase1 (*P. aeruginosa* ATCC 15692/PAO1)	DLDH1_PSEAE	160	7	R|AMAQGEMVAELIAGK<br>R|AMTLEANEGFVR<br>K|VISSTEALAPGSLPK<br>K|AVSELSTAFAQSLEMGA<br>R R|EIAADQVLVAVGR<br>R|SALGIQVQAPSIDIAR<br>K|SVAVELAGGGSQR	25	I	48.6
Aldehyde dehydrogenase *(Pseudomonas oleovorans)*	ALDH_PSEOL	34	4	K|FNEILVKEIVR<br>K|GAKILQGGQVDATE<br>R R|RSADYCR<br>K|VKQLIGITVK	9	I	52.8
Dihydrolipoyllysine-residue succinyltransferase component of 2-oxoglutarate dehydrogenase complex (*P. aeruginosa* ATCC 15692/PAO1)	ODO2_PSEAE	50	3	K|APTFPESVADGTVATWHK<br>K|LAEEAGIDPNSIAGTGK<br>K|PIMDLR	10	I	42.9
Glycerol kinase (*P. aeruginosa* ATCC 15692/PAO1)	GLPK1_PSEAE	190	9	R|DGLEDYIR<br>R|DVLDAMQR<br>R|ETTGLVTDPYFSGTK<br>R|MLEVLDIPR<br>R|REKLYAGWKKAVERTR<br>R|SAAICAQLKR	22	I	55.9

				R|VFEPECDEPRR<br>K|VINDSFDSEYFATK<br>K|WILDNVEGAR|E			
Dihydrolipoyllysine-residue acetyltransferase component of pyruvate dehydrogenase complex (*P. aeruginosa* ATCC 15692/PAO1)	ODP2_PSEAE	32	3	K|ADTPAPVGAPSR<br>K|VGDEVGTGDLILK<br>K|ANVIEVMVK	9	I	56.7
**E-** Amino acid transport metabolism							
Arginine deiminase (*P. aeruginosa* ATCC 15692/PAO1)	ARCA_PSEAE	27	2	K|GAAERVIVAGLPK<br>R|GGGHCMTCPIVR	9	I	46.4
Ornithine carbamoyltransferase, catabolic (*P. aeruginosa* ATCC 15692/PAO1)	OTCC_PSEAE	68	4	R|ITLTEDPKEAVK<br>R|MYDAIEYR<br>R|NNMGNSLLLIGAK<br>K|YTGTEQQHLKR	13	I	38
N-acetyl-gamma-glutamyl-phosphate reductase (*P. aeruginosa* ATCC 15692/PAO1)	ARGC_PSEAE	30	2	R|HLPEISQGLRR<br>K|VGIVGGTGYTGVELLR		I	36.7
Glycine cleavage system H protein 1 (*P. aeruginosa* ATCC 15692/PAO1)	GCSH1_PSEAE	30	2	R|FRPADAGAWEK<br>K|LLDQAAYDR	16	II	13.7
Glycine cleavage system H protein 2 (*P. aeruginosa* ATCC 15692/PAO1)	GCSH2_PSEAE	28	2	K|LKPSNPAELDK<br>M|SNIPAELR	15	II	13.6
Glutaminase-asparaginase (*P. aeruginosa* ATCC 15692/PAO1)	ASPQ_PSEAE	32	2	K|LSNVVILATGGTIAGAGASA ANSATYTAAK<br>K|VPVDQLLASVPQLK	13	II	38.6
Histidine ammonia-lyase (*P. aeruginosa* ATCC 15692/PAO1)	HUTH_PSEAE	30	2	R|DLLTASSEVAR<br>R|TAYGINTGFGLLASTR	5	I	53.8

Leucine-, isoleucine-, valine-, threonine- and alanine-binding protein<br>(*P. aeruginosa* ATCC15692/PAO1)	BRAC_PSEAE	58	3	K|VAVFEGLNAGDKDFNALISK<br>R|TIGLDNMQGPVAGK<br>K|IALAGPVTGPVAQYGDMQR	23	I	39.7
Methylmalonate-semialdehyde dehydrogenase<br>(*P. aeruginosa* ATCC15692/PAO1)	MMSA_PSEAE	80	4	K|AQVISNLVGASVGAAGQR<br>K|GVLQVVHGGK<br>R|LAELFLEAGAPK<br>R|LINESPYGNGTSIFTSSGAAAR	12	I	53.5
**G-** Carbohydrate transport and metabolism							
Phosphoglycerate kinase<br>(*P. aeruginosa* ATCC 15692/PAO1)	PGK_PSEAE	64	4	K|EFAESAVATVK<br>K|SLYEADLVETAK<br>K|VAAAGPLLAAELDALGK<br>K|VLPAVEILEQR	16	II	40.4
Glycosyltransferase alg8<br>(*P. aeruginosa* ATCC 15692/PAO1)	ALG8_PSEAE	19	2	R|HINMCSMALSK<br>R|RLGWFTMLVLFDQR	5	I	56.5
Glucans biosynthesis glucosyltransferase H<br>(*P. aeruginosa* PA7)	OPGH_PSEA7	32	4	R|ELRATDEYTYENR<br>R|MQQFATR<br>R|SNPPKPK<br>K|VIPEPWR	4	I	96
**I-** Lipid transport and metabolism							
Acetyl-CoA acetyltransferase<br>(*P. aeruginosa* ATCC 15692/PAO1)	ATOB_PSEAE	20	2	-|MQDVVIVAATR<br>R|TGLRMGHAK	3	II	40.3
Acyl carrier protein 1<br>(*P. aeruginosa* ATCC 15692/PAO1)	ACP1_PSEAE	50	2	K|ITTVQEAIDYIVAHQQ<br>K|IVAEQLGVK	19	III	8.7
**H**- Coenzyme transport and metabolism							

L-aspartate oxidase (*P. aeruginosa* ATCC 15692/PAO1)	NADB_PSEAE	17	2	R|LGIDCVYLDISHK<br>K|RCLGAYVLNR	4	I	60
Glutathione reductase (*P. aeruginosa* ATCC 15692/PAO1)	GSHR_PSEAE	22	3	K|GLDLQFNSDIAR<br>K|IFESRFR<br>K|VKIFESR	5	I	49
**K**- Transcription							
Transcription elongation factor GreA (*P. aeruginosa* ATCC 15692/PAO1)	GREA_PSEAE	77	3	K|ISVNSPIAR<br>K|LSNAQVIDVTAIPHSGK<br>R|TPGGDVEYEIVEVR	25	I	17.1
DNA-binding protein HU-beta (*P. aeruginosa* ATCC 15692/PAO1)	DBHB_PSEAE	44	2	R|ALDAVIESVTGALK<br>K|SELIDAIAASADIPK	32	III	9
**L**- Replication and repair							
DNA polymerase III subunit beta (*P. aeruginosa* ATCC 15692/PAO1)	DPO3B_PSEAE	51	3	R|ATTGEFTFTSK<br>R|HQVIVPRK<br>R|TSFAMAQQDVR	8	I	40.6
**J-** Translation, ribosomal structure and biogenesis							
Elongation factor Tu (*P. aeruginosa* UCBPP-PA14)	EFTU_PSEAB	38	2	R|AGENVGILLR<br>K|FECEVYVLSK	5	III	43.3
50S ribosomal proteins L36 2 (*P. aeruginosa*)	RL362_PSEAB	42	3	R|FKCVQGR<br>R|HRDCQVVKRRGRLYVICKSN PR<br>-|MKVLASLKQAKLR	86	III	5.9
50S ribosomal proteins L 20 (*P. aeruginosa* ATCC15692/PAO1)	RL20_PSEAE	22	2	R|DRRQRKRQFR<br>K|RQFRALWIAR	13	III	13.3

30S ribosomal proteins S14	RS14_PSEAE	21	2	R|CRLTGRPHGFYR	21	III	11.5
(*P. aeruginosa* ATCC15692/PAO1)				R|NKLREAAMR			
50S ribosomal proteins L36	RL36_PSEAE	24	2	K|KLCRNCKIIRRDGIVR	58	III	4.4
(*P. aeruginosa* ATCC15692/PAO1)				K|VRASVKKLCRNCK			
**M-** Cell wall/membrane biogenesis							
Peptidoglycan-associated lipoprotein	PAL_PSEAE	68	3	R|EYNMALGER	31	II	15.8
(*P. aeruginosa* ATCC 15692/PAO1)				R|VVLEGHTDER			
				R|YLVLQGVSPAQLELVSYGK			
**N-** Cell motility							
Protein PilJ	PILJ_PSEAE	26	2	R|LAQDAGVALEEIEK	4	I	72.5
(*P. aeruginosa* ATCC15692/PAO1)				R|SSAATKQIEALVK			
Protein PilH	PILH_PSEAE	72	3	K|DAETSAIPVIIVTTK|D	23	II	13.2
(*P. aeruginosa* ATCC 15692/PAO1)				R|ILIVDDSPTEMYK|L			
				R|ILIVDDSPTEMYKLTAMLEK			
A-type flagellin [CHAIN 0]	FLICA_PSEAI	82	4	-|ALTVNTNIASLNTQR	13	II	39.9
(*P. aeruginosa*)				R|FDNTINNLK			
				R|NLNNSSASLNTSLQR			
				R|LTSQVNGLNVATK			
B-type flagellin	FLICB_PSEAE	30	2	M|ALTVNTNIASLNTQR	5	I	49
(*P. aeruginosa* ATCC15692/PAO1)				K|EVAAQQAELTR			
Flagellar motor switch protein FliG	FLIG_PSEAE	28	3	K|EILTIARR	9	II	37
(*P. aeruginosa* ATCC15692/PAO1)				R|KMLTQALGEDK			
				R|EKVFKNMSKR|A			
**O-** Posttranslational modification, protein turnover, chaperones							
Thiol:disulfide interchange protein DsbA	DSBA_PSEAB	35	2	R|FDIGSAGGPEETLK	12	II	23.3
(*P. aeruginosa*, UCBPP-PA14)				K|LADYLIEK			
Thioredoxin	THIO_PSEAE	86	4	K|LNIDENQDTPPK	43	II	11.8

(*P. aeruginosa* ATCC 15692/PAO1)				K|MIAPVLDEVAR<br>K|SQLAAFLDANI<br>M|SEHIVNVTDASFEQDVLK			
Chaperone protein DnaK<br>(*P. aeruginosa* ATCC 15692/PAO1)	DNAK_PSEAB	123	6	K|ALGELEAAVK<br>K|ASSGLSEDEIQQMVR<br>K|FDLADIPPAPR<br>K|MNALSQASTPLAQK<br>R|NQGDALVHATR<br>R|RFEENVVQK	11	I	68.3
Alkyl hydroperoxide reductase subunit, Thioredoxin peroxidase<br>(*P. aeruginosa* ATCC 15692/PAO1)	AHPF_PSEAE	22	3	K|AKGVCFCPHCDGPLFK<br>K|GVCFCPHCDGPLFK<br>R|HYEVDIMNLQR	12	II	20.5

[a]According to https://www.ncbi.nlm.nih.gov/COG/

[b]Fraction in size exclusion chromatography

Table 3. Proteins differentially expressed in *Pseudomonas aeruginosa* grown in LB without addition of cadmium (control), classified according to COG[a] and identified on the basis of at least two matched peptides and FDR 1%.

Identified protein (strain, species)	Entry name	EasyProt score	Number of matched peptides	Sequence coverage, %	Peptide sequence	Molecular mass, kDa	COG[a]	Fraction[b]
tRNA (guanine-N(1)-)-methyltransferase	TRMD_PSEAE	29	3	13	K|RVPEVLLSGNHEHIRR<br>R|PEVYADKR	28.4	J	I

(*P. aeruginosa* ATCC 15692/PAO1)					K|LLAEYIRQR			
Ribosomal RNA large subunit methyltransferase G (*P. aeruginosa* PA7)	RLMG_PSEA7	28	3	10	K|NLARNGLDLGSVR R|PAPRSPYPTRYR K|TLALLEEQLIR	40.5	J	I
DNA-directed RNA polymerase subunit beta' (RNAP subunit beta') (*P. mendoncina*)	RPOC_PSEMY	27	4	3	R|IVDTTVGR K|KMALELFKPFIFGKLE MR R|KRKRDADKPVR R|RGRAITGSNKR	154.7	K	I
Probable Fe(2+)-trafficking protein (*P. aeruginosa* ATCC 15692/PAO1)	FETP_PSEAE	21	2	21	R|LNMMNAEDR -|MSRTVMCRK	10.6	P	I
Regulatory protein NosR	NOSR_PSEAI	21	2	30	- |CRYICPLGAALAIPSKFR R|KECGNPCQLCAK	11.3	K/L	I
tRNA (mo5U34)-methyltransferase (*P. mendoncina*)	CMOB_PSEMY	18	2	9	R|WLRRAGFEDVR R|YAQMRNVWFLPSVPA LER	36.3	J	I
Protein phosphatase CheZ (*P. aeruginosa* ATCC 15692/PAO1)	CHEZ_PSEAE	17	2	10	R|ELHQEWQRFMRR M|QLIQELSQAR	25.7	T	I
3-hydroxydecanoyl-[acyl-carrier-protein] dehydratase (*P. mendoncina*)	FABA_PSEMY	19	2	16	K|KVTYNIHIKR - |MTRQNAYTREDLLACS R	18.7	I	I
NADH pyrophosphatase (*P. aeruginosa)*	NUDC_PSEAB	19	2	9	R|HNRFCGNCGTR R|VMQCPQCGLHQYPR	31.3	H	I
ATP synthase gamma chain (*P. aeruginosa* ATCC 15692/PAO1)	ATPG_PSEAE	18	2	8	R|GLCGGLNINLFK R|HPFMVEREVKR	31.6	C	I

UPF0176 protein Pmen_1693 (*P. mendoncina*)	Y1693_PSEMY	18	2	11	R|CEKASSYMLGEGFEEVYHLKGGILK<br>K|SFREFPEYVK	35.5	/	I
UTP--glucose-1-phosphate uridylyltransferase (UDPGP) (*P. aeruginosa* ATCC 15692/PAO1)	GALU_PSEAE	18	2	12	R|FLPATKAMPKEMLPVVNK<br>K|YGVIAGEMIRDDIFR	31.2	C	I
Type III pantothenate kinase (*P. mendoncina*)	COAX_PSEMY	17	2	9	R|CRLVSVR<br>-|MILELDCGNSFIKWR	26.7	H	I
Phenazine biosynthesis protein phzA 2 (*P. aeruginosa* ATCC 15692/PAO1)	PHZA2_PSEAE	27	3	12	R|EYQRLK<br>-|MREYQRLK<br>R|NREFMNPMQKLR	19.3	Q	I
Nuclease sbcCD subunit C (*P. aeruginosa* ATCC 15692/PAO1)	SBCC_PSEAE	19	3	3	R|ERQAQADNHLR<br>R|QQTLEAER<br>R|QRQQDEFQRLQADWQAWR	139.0	L	I
Lipoprotein-releasing system ATP-binding protein LolD (*P. aeruginosa* ATCC 15692/PAO1)	LOLD_PSEAE	18	2	11	-|MNDKSVLSCR<br>R|QRAAELLERVGLGHR	24.7	M	I
Putative quercetin 2,3-dioxygenase PA1205 (Putative quercetinase) (*P. aeruginosa* ATCC 15692/PAO1)	Y1205_PSEAE	18	2	10	R|DCPERHEHLLQR<br>R|FGRLDALEGPRLSAPPIPWK	24.4	R	I
tRNA sulfurtransferase (*P. mendoncina*)	THII_PSEMY	17	2	5	K|LIVKVFPEITIK<br>R|QECNAAGIDLKK	54.7	J	I
Peptide chain release factor 2 (RF-2) (*P. aeruginosa* ATCC 15692/PAO1)	RF2_PSEAE	36	4	11	R|GRRRGRRRR<br>R|LRRHYHRAVRGR<br>R|PQRLEQPR<br>R|PQRLEQPRIRPEPRPRA	41.20	J	II

<table>
<tr><td></td><td></td><td></td><td></td><td></td><td>R</td><td></td><td></td><td></td></tr>
<tr><td>Protein sprT
(P. aeruginosa PA7)</td><td>SPRT_PSEA7</td><td>35</td><td>3</td><td>21</td><td>K|GRRYYCR
-|MPEHLNARVEACYR
R|RCKATLVFSGEVTR</td><td>19.7</td><td>P</td><td>II</td></tr>
<tr><td>Outer membrane porin F [CHAIN 0]
(P. aeruginosa ATCC 15692/PAO1)</td><td>PORF_PSEAE</td><td>28</td><td>2</td><td>7</td><td>R|DVLVNEYGVEGGR
K|SKVKENSYADIK</td><td>35.2</td><td>M</td><td>II</td></tr>
<tr><td>Translation initiation factor IF-2
(P. aeruginosa ATCC 15692/PAO1)</td><td>IF2_PSEAE</td><td>23</td><td>3</td><td>5</td><td>K|KTYVKRSPDEIEAERQR
K|PAAVEERKKEEPRRVPK
K|TVSVEVRKK</td><td>90.9</td><td>J</td><td>II</td></tr>
<tr><td>30S ribosomal protein S13
(P. aeruginosa ATCC 15692/PAO1)</td><td>RS13_PSEAE</td><td>22</td><td>2</td><td>14</td><td>R|HRRGLPVR
K|ITTEGDLR</td><td>13.2</td><td>J</td><td>II</td></tr>
<tr><td>Uncharacterized protein PA0525
(P. aeruginosa ATCC 15692/PAO1)</td><td>Y525_PSEAE</td><td>22</td><td>3</td><td>4</td><td>R|LLQLYR
R|RASQVLGGQPQKR
R|RQFACLR</td><td>69.47</td><td>/</td><td>II</td></tr>
<tr><td>SsrA-binding protein
(P. aeruginosa ATCC 15692/PAO1)</td><td>SSRP_PSEAE</td><td>18</td><td>2</td><td>14</td><td>K|GYACVALSMYWKK
R|HTEKERDSDR</td><td>18.0</td><td>J</td><td>II</td></tr>
<tr><td>30S ribosomal protein S3
(P. aeruginosa ATCC 15692/PAO1)</td><td>RS3_PSEAE</td><td>18</td><td>2</td><td>15</td><td>-|MGQKVHPNGIRLGIVK
K|PELDAMLVAQSVAQQLER</td><td>25.8</td><td>J</td><td>II</td></tr>
<tr><td>Copper-binding periplasmic protein [CHAIN 0]
(P. aeruginosa ATCC 15692/PAO1)</td><td>NOSD_PSEAE</td><td>17</td><td>2</td><td>6</td><td>R|DSHPLMRMPAAEPRP
-|EPVDGLPLR</td><td>43.9</td><td>P</td><td>II</td></tr>
<tr><td>UPF0178 protein PLES_56411
(P. aeruginosa, strain LESB58)</td><td>Y5641_PSEA8</td><td>20</td><td>2</td><td>14</td><td>R|IWIDADACPKVAKELVCK</td><td>17.8</td><td>/</td><td>II</td></tr>
</table>

					M|RIWIDADACPKVAKE LVCKFALK			
Transcriptional regulatory protein AlgQ (*P. aeruginosa* ATCC 15692/PAO1)	ALGQ_PSEAE	18	2	8	R|CDNGDCR -|MLESCR	18.0	K	II
GTPase Der (*P. aeruginosa* ATCC 15692/PAO1)	DER_PSEAE	17	2	8	K|STLFNRLTR K|YTLIDTAGVRRRGKIF EAVEKFSVVK	55.0	R	II
50S ribosomal protein L31 (*P. aeruginosa* ATCC 15692/PAO1)	RL31_PSEAE	20	2	23	K|QKVLDTGGR K|TRSTLCK	7.9	J	II
50S ribosomal protein L18 (*P. mendoncina*)	RL18_PSEMY	20	2	22	R|LKMHELEAVRLCVYR -|MTDKKVTRLRR|A	12.6	J	II
Beta-lactamase OXA-15 [CHAIN 0]	BLO15_PSEAI	19	2	12	R|AMLVFDPVRSKKR K|LYRNELPFRVEHQRLV K	29.3	V	II
Pyridoxine/pyridoxamine 5'-phosphate oxidase (PNPOx) (*P. aeruginosa)*	PDXH_PSEAB	19	2	13	R|AELERLLADTERR R|LHDRLDYRRQDGGWS R	24.8	H	II

[a]According to https://www.ncbi.nlm.nih.gov/COG/

[b]Fraction in size exclusion chromatography

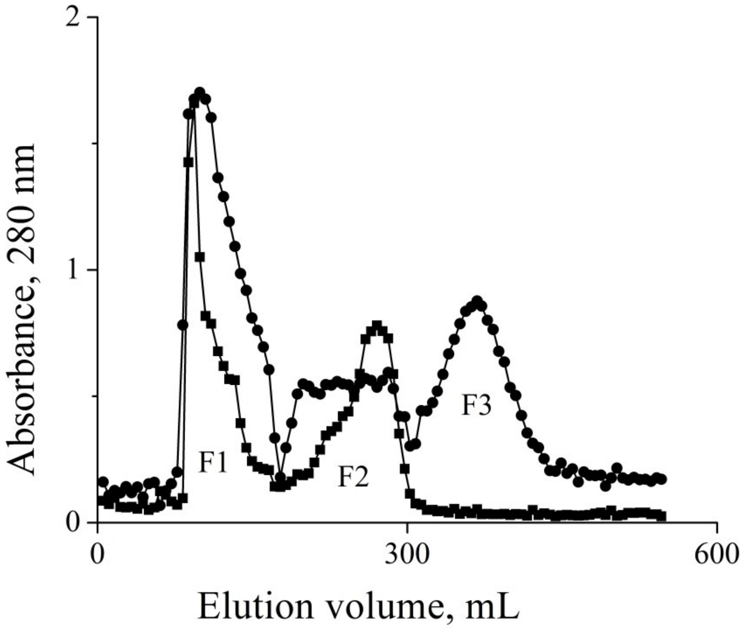

Figure 1. Size exclusion chromatography on Sephadex G-100 column (2.5 x 70 cm) equilibrated in buffer A (50 mM Tris buffer pH 7.5 supplemented with 0.1 mM PMSF and 0.5 mM DTT). Fractions (volumes of 5.5 mL) were collected and absorbance at 280 nm was recorded. Fractions were pooled according to absorbance. ■ - control, ●- cadmium amended biomass
